# Eagle’s Syndrome as a Cause of Discomfort and the Subjective Presence of a Foreign Body in the Throat

**DOI:** 10.3390/diagnostics11101832

**Published:** 2021-10-03

**Authors:** Irena Wolińska, Przemysław Jaźwiec, Maria Pawłowska, Paweł Gać, Rafał Poręba, Małgorzata Poręba

**Affiliations:** 1 Department of Pathophysiology, Wroclaw Medical University, Marcinkowskiego 1, 50-368 Wrocław, Poland; irena.wolinska@student.umed.wroc.pl; 2 Specialist Medical Center in Polanica-Zdrój, Jana Pawła II 2, 57-320 Polanica Zdrój, Poland; przemkolog@wp.pl (P.J.); maria.pawlowska@yahoo.pl (M.P.); 3 Department of Hygiene, Wroclaw Medical University, Mikulicza-Radeckiego 7, 50-368 Wrocław, Poland; 4 Department of Internal Medicine, Occupational Diseases and Hypertension, Wroclaw Medical University, Borowska 213, 50-556 Wrocław, Poland; rafal.poreba@umed.wroc.pl

**Keywords:** Eagle syndrome, styloid process, stylohyoid ligament

## Abstract

Eagle syndrome consists of symptoms resulting from the elongation and excessive calcification of the styloid process of the temporal bone and calcification of the ligaments associated with this process. The main symptoms of this syndrome are the feeling of a foreign body in the throat, dysphagia and pain localized in the temporomandibular region, neck and ear. The authors describe the case report of a previously healthy 39-year-old Caucasian male that complained of discomfort and foreign body sensation in his throat. Computed tomography (CT) showed the presence of an elongated styloid process bilaterally with clear predomination at the left side. The patient underwent laryngological and surgical consultation. Due to the lack of symptoms related to the compression of the carotid arteries, no surgery was recommended. In summary, Eagle’s syndrome is a rare condition characterized by craniofacial pain or foreign body sensation that should be considered, especially if the pain is unilateral. CT imaging in his case was a perfect tool and enabled a suitable diagnosis of this rare syndrome.

## 1. Introduction

The first to report a clinical case of elongated styloid process was Weinlecher in 1872, but it was Watt W. Eagle in 1947 who described a set of symptoms resulting from the lengthening and excessive calcification of the styloid process of the temporal bone and calcification of the ligaments attaching to this process, i.e., the styloid and stylomandibular ligaments [1,2,3]. This abnormality is found in around 4% of the population, but for most cases, it is asymptomatic. The main symptom is the pain located in the temporomandibular area, neck, and ear. Various head movements might trigger it [4,5]. Patients may also feel a foreign body sensation in their throat, dysphagia, tinnitus, and balance disorders. The most dangerous symptoms are direct trauma of the elongated styloid process to the internal carotid artery, including epileptic attacks and stroke [5,6]. There is a possibility to treat patients with both non-invasive and invasive treatment, but surgery is the treatment of choice with the highest success rate [5,7].

We describe the case of a 39-year-old man who presented with discomfort and foreign body sensation in his throat.

## 2. Case Description

The patient was a Caucasian male aged 39, who reported to a neurologist because of his throat’s discomfort and foreign body sensation. The throat discomfort had been occurring periodically for many years. The throat discomfort and the foreign body sensation intensified following an episode of vomiting several weeks prior to the visit. The vomiting episode was related to a dietary error. He denied odynophagia, dysphagia, tinnitus, vision disturbances, headaches, and relevant risk factors or medication. He had an otherwise healthy life before the event, reporting no invasive procedures at the area of the throat and larynx, cervical part of spine as well as cranial area. In his childhood, he was only diagnosed with thyroid nontoxic goiter.

On physical examination, head movements or opening the mouth do not cause pain, but currently, there is pressure soreness in the submandibular area on the left side. There were no neurological deficits.

The neck ultrasound did not show any enlarged lymph nodes or nodal packages.

**Figure 1:**
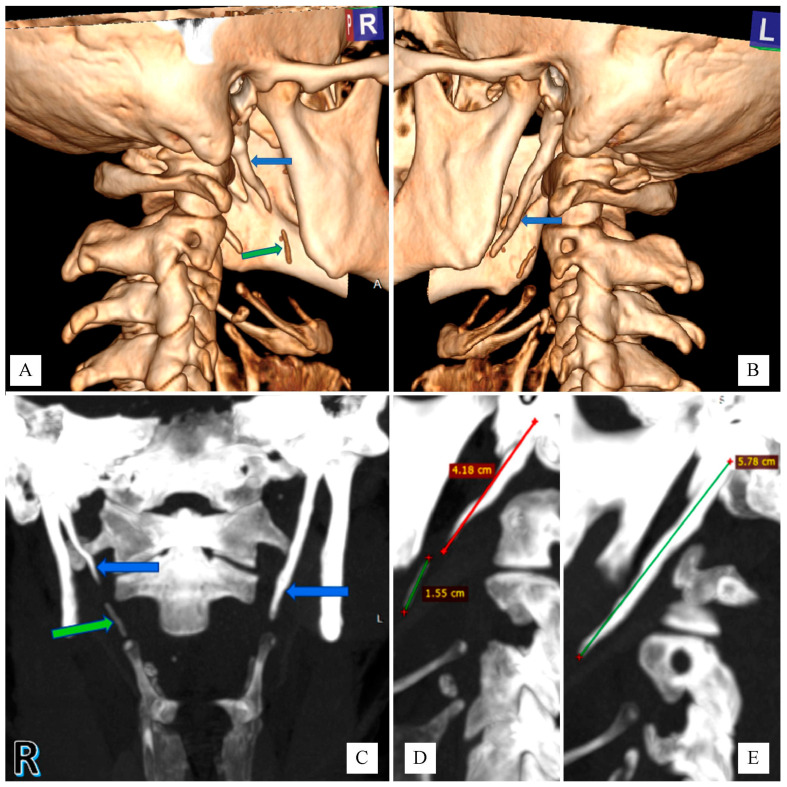
Computed tomography of the neck without contrast agent: (**A**). Volume Rendering Technique (VRT) reconstruction. The elongated right styloid process is marked with a blue arrow. Longitudinal calcification in the extension of the right styloid process is marked by a green arrow. (**B**). VRT reconstruction. The elongated left styloid process is marked with a blue arrow. (**C**). Maximum intensity projection (MIP) reconstruction. Elongated styloid processes are marked with a blue arrow. Longitudinal calcification in the extension of the right styloid process is marked by a green arrow. (**D**). MIP reconstruction. Measurements of the length of the elongated right styloid process and calcification in its extension. (**E**). MIP reconstruction. Measurement of the length of the elongated left styloid process.

The computed tomography (CT) scan showed a bilaterally elongated styloid process: 57–58 mm on the left side, and the right side was 41–42 mm (Figure 1). Below the top of the right styloid process, there was placed a well-calcified shadow about 15–16 mm long, which could correspond to its continuation, without a clear connection with the top of the process (Figure 1). In addition, the study showed adenoid tissue overgrowth of Waldeyer’s lymph ring with the presence of minor polycyclic calcifications, abolition of physiological cervical lordosis and degenerative changes in the cervical spine.

Eventually, the diagnosis of the classic type of Eagle’s syndrome (ES) was made; on the left side, so called elongated type, and on the right-side the segmented type (consisting of an uninterrupted segments of mineralized ligament). The patient underwent laryngological and surgical consultation. Due to the lack of symptoms related to the compression of the carotid arteries, no surgery was recommended. Pharmacological treatment was prescribed—first of all, painkillers. In the event of persistent symptoms, it was recommended to consider the inclusion of steroid and antiepileptic drugs in the treatment. The patient is under follow-up. An evaluation of the effectiveness of treatment with painkillers has been planned.

## 3. Discussion

The styloid process is a bony structure coming out from the temporal bone anteriorly from the stylomastoid foramen. It is clinically significant because of the structures surrounding it, and especially because the tip of the styloid process is located close to the external carotid artery laterally and also it has proximity to the internal carotid artery, the internal jugular vein, the jugular part of the sympathetic trunk and the V, VII and IX-XII cranial nerves [8,9].

The etiopathogenesis of Eagle’s syndrome has not been fully elucidated, but it is associated with the compression of the elongated styloid process on the above-mentioned cranial nerves. In some cases, it was diagnosed after tonsillectomy or after direct trauma [5,10]. Dental procedures are also suspected to be the cause of iatrogenic ES [11].

It is assumed that the styloid process measures 2.5–3 cm in length; when it surpasses 3 cm, it is elongated. Nevertheless, some analysis shows that in around 15% of cases shorter styloid process might also cause ES symports [2,5].

There are two main types of Eagle’s syndrome—the “classic type” and the “stylo-carotid artery type” [8,12]. The first one presents typical symptoms such as a persistent sore throat with a foreign body sensation, dysphagia, taste disturbances, change in tone of voice or trismus.

The stylo-carotid artery type symptoms are caused by the pressure of the styloid process on the jugular vessels. Pressure on the internal carotid artery can lead to pain radiating along the artery’s course, while pressure on the external carotid artery causes facial pain.

Compression-induced blood flow restriction in the internal carotid artery can cause neurological disorders, ranging from nausea and fainting to aphasia and visual disturbances. There are several cases of bilateral carotid dissection, ischemic strokes secondary to Eagle’s syndrome [3,5,13].

An elongated styloid process might cause venous reflux obstruction and, in effect, internal jugular stenosis [14,15].

The diagnosis of Eagle’s syndrome is usually based on a physical examination by palpation of the elongated styloid process and radiographs. The X-ray testing of the styloid process is enough to determine the length of the process; however, CT enables the precise evaluating of the anatomical relationship between the process and surrounding structures, such as nerves and blood vessels and now is treated as a “gold standard” [16,17]. It is accepted that multiple row CT and 3D reconstruction is the best method of diagnosing Eagle syndrome [5,18,19].

Treatment for Eagle syndrome includes non-invasive treatment and surgery, and it depends on the patient’s symptoms. Non-invasive treatment includes painkillers, injecting steroids and/or antiepileptic drugs. Surgical treatment can shorten the elongated styloid process by oral or external access [5,15,16,19,20]. In this case, the patient is treated with painkillers. The evaluation of the effectiveness of the treatment is planned.

## 4. Conclusions

Eagle syndrome is a rare clinical condition that is associated with styloid process elongation. Imaging diagnostics like computed tomography is a valuable tool for diagnosis and implementation of appropriate treatment.

## Data Availability

The study did not provide any data that could be used as a database.
